# Do endocannabinoids acting via hepatic CB-1 contribute to NAFLD and hepatic insulin resistance? 

**DOI:** 10.1172/JCI155330

**Published:** 2022-01-04

**Authors:** George Kunos, Tony Jourdan, Joseph Tam

**Affiliations:** 1 Laboratory of Physiologic Studies, National Institute on Alcohol Abuse and Alcoholism (NIAAA), NIH, Bethesda, Maryland, USA.; 2 Team Pathophysiology of Dyslipidemia, INSERM UMR1231 “Lipids, Nutrition, Cancer” and Université de Bourgogne Franche-Comté, Dijon, France.; 3 Obesity and Metabolism Laboratory, Institute for Drug Research, School of Pharmacy, Faculty of Medicine, The Hebrew University of Jerusalem, Jerusalem, Israel.

**Keywords:** Metabolism, G protein&ndash;coupled receptors, Insulin signaling, Mouse models

## To the Editor:

The article by Wang et al. (1) challenges the concept, proposed and developed by us, that endocannabinoids acting via hepatic cannabinoid receptor 1 (CB-1) contribute to nonalcoholic fatty liver disease (NAFLD) and related hepatic insulin resistance. Our rebuttal is based on several points.

In three published studies using genetically altered mice to explore the role of hepatocyte CB-1 in NAFLD, we used a diet rich in saturated fat (TD97070), which increased hepatic anandamide levels (2), a critical factor in the increased signaling of hepatic CB-1 in NAFLD. In contrast, the D12492 diet used by the authors (1) has less saturated fat and much larger amounts of monounsaturated fat acids (MUFAs) and polyunsaturated fatty acids (PUFAs), and it did not affect hepatic anandamide in a side-by-side comparison of these two diets (3). Hepatic CB-1 is autoinduced by its endogenous ligand (4), which may be one reason why D12492 did not induce hepatic CB-1 expression (1), although the authors’ use of C57Bl6/N mice instead of the C57Bl6/J mice we used may also be a factor. Furthermore, the paltry weight gain (10 g over chow-fed mice) and minimal diet-induced whole-body insulin resistance (Supplemental Figure 3 in ref. 1) may have been insufficient to induce the hepatic endocannabinoid system, as a robust obesity-related upregulation of the CB1b isoform in human liver was observed only in individuals with a BMI above 30 (5).

Documenting organ-specific insulin resistance requires the use of hyperinsulinemic/euglycemic clamps, which showed that a high-fat diet (HFD) elicits CB-1–mediated hepatic insulin resistance due to increased glycogenolysis (2). CB-1 is functional even in healthy liver, as indicated by CB-1–mediated inhibition of insulin-induced Akt phosphorylation in primary hepatocytes from lean mice (2). Neither approach has been used or discussed by the authors.

Information obtained using hepatocyte-specific *Cnr1^–/–^* mice should be verified using a rescue model in which *Cnr1* is reexpressed in hepatocytes of global *Cnr1^–/–^* mice. Using such a model reinforced our conclusions of a partial contribution of hepatocyte CB-1 to NAFLD and insulin resistance (2); the authors did not utilize such a model. The phenotype of *hCnr1^–/–^* mice should have been further confirmed by pharmacological antagonism using a peripheral CB-1 blocker. In numerous studies, including our own, this resulted in near-complete reversal of HFD-induced steatosis and insulin resistance. Pharmacological blockade was not tested by the authors.

Finally, the authors conspicuously do not cite any of the multiple human and animal studies that are consistent with the role of endocannabinoids and hepatocyte CB-1 in NAFLD and hepatic insulin resistance.

## Version 1 01/04/2022

Electronic publication
